# The complete mitochondrial genome of *Pseudorasbora interrupta* and phylogeny of *Pseudorasbora*

**DOI:** 10.1080/23802359.2019.1673229

**Published:** 2019-10-01

**Authors:** Chao Li, Hung-Du Lin, Jun Zhao

**Affiliations:** a Guangzhou Key Laboratory of Subtropical Biodiversity and Biomonitoring, Guangdong Provincial Key Laboratory for Healthy and Safe Aquaculture, Guangdong Provincial Engineering Technology Research Center for Environmentally-friendly Aquaculture, School of Life Science, South China Normal University, Guangzhou, China;; b Department of Biology, The Affiliated School of National Tainan First Senior High School, Tainan, People's Republic of China

**Keywords:** Complete mitochondrial genome, *Pseudorasbora interrupta*, *Pseudorasbora parva*, Gobioninae, phylogeny

## Abstract

We sequenced the mitochondrial genome of *Pseudorasbora interrupta* using Illumina technology and additional Sanger sequencing. The assembled 16601 bp mitogenome had a GC content of 40.98% and consisted of 13 protein-coding genes, 22 tRNA genes, two rRNA genes, and one non-coding control region (CR), with a gene order identical to the fishes. In addition, we downloaded the mitogenome of the closely related species Topmouth Gudgeon *P. parva*. The mitogenomes of *P. interrupta* and *P. parva* showed a sequence identity of 99.2% with the previously published *P. parva* mitogenome. The phylogenetic reconstruction based on 14 Gobioninae mitogenomes supported *P. interrupta* and its sister species *P. parva* as a monophyletic group. However, *Pseudorasbora* was proved to be a polyphyletic group which means amendments will be needed for the taxonomy of this genus.

*Pseudorasbora interrupta* (Cyprinidae, Cypriniformes) was erected as novel species in 2007 (Xiao et al., 2007). It is only distributed in Fenghuang Mountain, Chaozhou City, Guangdong Province, China. This species can be distinguished by several characters from its close related species *P. parva*, which distributed in East Asia, such as an incomplete lateral line scales, a lower body depth which is always shorter than head length and a weak lateral process of maxilla bone. Moreover, it also resembles *P. pumila* distributed in Japan with incomplete lateral line scales, but differs from the latter in the number of lateral line scales with 7–15 vs. 3–5. Since it was described, there are few research about it due to sampling difficulty. *P. interrupta* showed a very close relationship with *P. parva* and together formed sister group with *P. pumila* according to nuclear gene sequences (Kim et al., 2013), which is consistent with their similarity in morphological features.

In order to identify *P. interrupta* and *P. parva* in the mitogenome level and help reconstructing the genus *Pseudorasbora* phylogenetic relationships, we sequenced the complete mitochondrial genome of *P. interrupta*.

An adult male *P. interrupta* specimen was collected from Fenghuang Mountain, Chaozhou City (geospatial coordinates: 23°55′N, 116°36′E) in May 2018 with net. The specimen is stored in Fish Collection Room, School of Life Science, South China Normal University, Guangzhou, China (Accession number: SCNU2018050001). Whole mitochondrial genome sequencing was done using Illumina X-ten platform performed in BGI (BGI Inc., Shenzhen, China).

The length was determined to be 16 601 bp. The overall base composition is 28.31% T, 24.53% C, 30.70% A and 16.45% G, and the GC content is relatively low 40.98%, very similar to the mitogenome of *P. parva* (GenBank accession number: JF802126), which contains 41.00% GC. Gene content contains 13 protein-coding genes, two ribosomal RNA genes, and 22 transfer RNA genes and a major non-coding control region (D-loop region). The mitochondrion sequence data were deposited to GenBank with accession No.MN175390.

Compared with the mitogenome of *P. parva*, an overall 0.8% divergence was revealed and 129 variable sites was detected in *P. interrupta*, indicating their close relationship. Hereto, molecular evidence of whole mitogenome showed that it is hard to distinguish *P. interrupta* and *P. parva*, combining with previous proof from morphology (Fan 2011) and feeding habits (Zhao et al., 2018), the status of species of *P. interrupta* should be reappraised as soon as possible. In the phylogenetic tree, *P. interrupta* grouped closely with *P. parva* and then formed sister clade with *P. pumila* (Figure 1). It is noticed that *P. elongata* didn’t cluster firstly with any species above but with *Pseudopungtungia tenuicorpus* (Figure 1), showing a relatively long distance with the remaining species of the genus *Pseudorasbora*. A conclusion was made that *Pseudorasbora* is a polyphyletic group which means amendments will be needed for the taxonomy of this genus. More morphological research will be needed for the correct classification of the genus.

**Figure 1.**
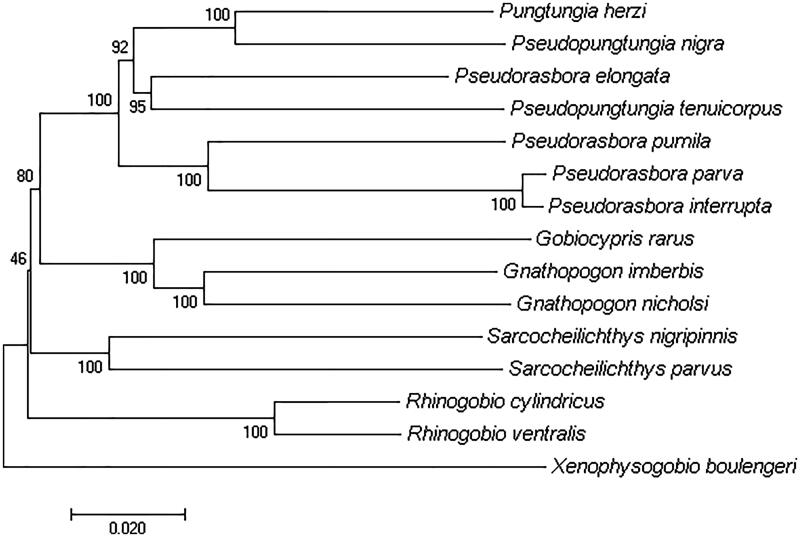
Phylogenetic tree of the complete mitochondrial genomes of 14 Gobioninae species including *P. parva* (JF802126), *P. interrupta* (MN175390), *P. pumila* (AB239599), *P. elongata* (NC_021769), *Pungtungia herzi* (KF006339), *Pseudopungtungia nigra* (NC_011161), *Pseudopungtungia tenuicorpus* (NC_014873), *Sarcocheilichthys nigripinnis* (NC_020608), *Sarcocheilichthys parvus* (NC_018786), *Gnathopogon imberbis* (NC_027255), *Gnathopogon nicholsi* (NC_033351), *Gobiocypris rarus* (NC_018099), *Rhinogobio cylindricus* (KU379652), *Rhinogobio ventralis* (KU379653).
